# Effect of the COVID-19 Lockdown on Ambient Air Quality in Major Cities of Nepal

**DOI:** 10.5696/2156-9614-11.29.210211

**Published:** 2021-03-02

**Authors:** Bashu Dev Baral, Kapil Thapa

**Affiliations:** Department of Zoology, Tri-Chandra Multiple Campus, Tribhuvan University, Kathmandu, Nepal

**Keywords:** Air Quality Index, AQI, COVID-19, lockdown, particulate matter

## Abstract

**Background.:**

The Nepalese government announced a nationwide lockdown beginning on March 24, 2020 as an attempt to restrain the spread of COVID-19. The prohibition in flight operations and movement of vehicles, factory shutdowns and restriction in people's movement due to the lockdown led to a significant reduction in the amounts of pollutants degrading air quality in many countries.

**Objectives.:**

The present study aimed to analyze changes in particulate matter (PM) emissions and the air quality index (AQI) of six cities in Nepal i.e., Damak, Simara, Kathmandu, Pokhara, Nepalgunj and Surkhet due to the nationwide lockdown in response to the COVID-19 outbreak.

**Methods.:**

Daily PM concentrations of each of the six study cities from January 24 to September 21, 2020 were obtained from the World Air Quality Index project (https://aqicn.org) and analyzed using R Studio software. The drop percentage was calculated to determine the change in PM_2.5_ and PM_10_ concentration during different time periods. Independent sample Mann–Whitney U tests were performed to test the significance of differences in mean concentration for each site during the lockdown period (24 March–24 July 2020) and its corresponding period in 2019. Similarly, the significance of differences in mean concentrations between the lockdown period and the period immediately before lockdown (23 January–23 March) was also examined using the same test.

**Results.:**

During the lockdown period, in overall Nepal, AQI_PM2.5_ and AQI_PM10_ were within the moderate zone for the maximum number of days. As a result of the lockdown, the highest immediate and final drop of PM_2.5_ was observed in Damak (26.37%) and Nepalgunj (80.86%), respectively. Similarly, the highest immediate drop of PM_10_ was observed in Surkhet (37.22%) and finally in Nepalgunj (81.14%). Analysis with the Mann–Whitney U test indicated that for both PM types, all sites showed a statistically significant (p < 0.05) difference in mean concentrations during lockdown and the corresponding period in 2019.

**Conclusions.:**

The present study explored the positive association between vehicular movement and PM emissions, highlighting the need for alternative fuel sources to improve air quality and human health.

**Competing Interests.:**

The authors declare no competing financial interests.

## Introduction

As the SARS-CoV-2, a novel coronavirus causing COVID-19 rapidly spread, the Director General of World Health Organization (WHO) declared the outbreak to be a Public Health Emergency of International Concern on 30 January 2020.^1^ The disease being air-borne and highly infectious,^2^ its outbreak ushered many countries to go into lockdown and restrict transportation, unnecessary gatherings and travel in order to contain virus spread.^3^ The government of Nepal also imposed a complete nationwide lockdown from 24 March 2020 after a second coronavirus case was confirmed, which restricted domestic and international flights, vehicular movement except for essential purposes, prohibited gatherings, suspended school and colleges and shut down factories, industries and brick kilns.^4^ Concerned about the suffering economy, the government lifted the nationwide lockdown on 22 July 2020 after almost 4 months. The country then underwent episodes of regional lockdowns in response to increased infection rates in some regions.

Although the efficacy of lockdown in Nepal to suppress the pandemic was minimal, it played an important role in improving air quality.^5–7^ Lockdown-induced improvement in air quality has also been reported in India,^8,9^ China,^8,10^ Pakistan,^11^ Iraq,^12^ and in other major global cities like Seoul, Los Angeles, London and Madrid.^13^ Improvements in air quality were inevitable as restrictions on automobiles and factories reduced anthropogenic emission of air pollutants.

The major pollutants affecting the atmosphere throughout the world are ozone, particulate matter (PM), lead (Pb), carbon monoxide (CO), nitrogen dioxide (NO_2_), sulfur dioxide (SO_2_), and other toxic compounds.^14^ In Nepal, the National Ambient Air Quality Standard (NAAQS) has defined nine variables, i.e., PM_2.5_, PM_10,_ ozone (O_3_), NO_2_, SO_2_, total suspended particulates (TSP), carbon monoxide (CO), benzene (C_6_H_6_), and lead (Pb) to characterize the air quality of a region. However, PM_2.5_ and PM_10_ are of primary concern as they are thought to be key contributors of air pollution.^15,16^

Particulate matter is a collective term describing small solid and liquid particles which are present in the atmosphere over relatively brief (minutes) to extended periods of time (days to weeks).^17^ The major sources of PM include emissions from power plants, industries, automobiles, construction sites, unpaved roads, fields, smokestacks and fires.^18^ Particles with a diameter greater than 10 μm generally do not get passed through nasal hairs and defense mechanisms of the upper respiratory system and are not of public health concern.^17^ However, PM less than 10 μm (PM_2.5_ and PM_10_) is of highest importance as it poses the most serious risks to human health.^15,19,20^ Epidemiological studies have shown positive associations between exposure to PM and frequency of certain illnesses and mortality. Exposure to fine PM is associated with a number of cardiovascular outcomes such as hypertension, atherosclerosis, arrhythmias, myocardial ischemia, heart attacks, heart failure and strokes.^20^ Karki *et al.* (2016) reported 11 300 inpatient admissions due to respiratory problems and a 3.7% rise in mortality and 1% rise in respiratory hospitalization per 10 μg/m^3^ rise in PM_2.5_. Similarly, a 10 μg/m^3^ increase in PM_10_ was found to increase risk of hospitalization by 1.70% for respiratory and 2.29% for cardiovascular admissions.^19^

**Abbreviations**
*AQI*: Air quality index
*NAAQS*: National Ambient Air Quality Standard
*USEPA*: United States Environmental Protection Agency
*WHO*: World Health Organization

A significant increase in the number of automobiles has created an alarming air pollution problem in Nepal, especially in densely populated cities like the Kathmandu valley.^22^ In fiscal year 2018–19, 318 477 new vehicles were registered, of which 78% were motorcycles and 6% were cars/vans.^23^ Similarly, within the same fiscal year, 436 new industries were registered, of which 38% were energy based, construction, manufacturing and mineral industries whose contributions to emissions are considered to be the major cause for the degradation of air quality.^24^ Nepal was ranked 8th in PM_2.5_ concentrations in the regional ranking of Central and South Asia, and the capital city of Nepal, Kathmandu was ranked as the sixth most polluted capital city.^25^ In 2020, the Environmental Performance Index of Nepal ranked it in the 178^th^ position in global air quality among 180 countries.^26^

Amidst the context of worsening air pollution, but continuous failure of most of governing bodies worldwide to address this environmental issue, the COVID-19 lockdown experience could be very beneficial for designing efficient pollution control measures. Studies have begun to quantify the efficiency of such lockdowns for controlling air pollution. Khan *et al.* (2020) studied the variation of air quality in major cities of Pakistan before and after the lockdown period.^11^ That study compared air quality in major cities before the lockdown from March 1 to March 23 and then during the lockdown from March 24 to April 15 and showed a 49% reduction in the amount of NO_2_ in Lahore, 45% in Peshawar and 56% in the twin-cities. In Nepal, Paudel *et al.* (2020) compared the concentration of PM_2.5_ in Ratnapark, Kathmandu during the lockdown period in 2020 with that of the corresponding period in 2019 to determine the impact of the lockdown on air quality.^7^ The results showed that the number of days exceeding the NAAQS of Nepal in 2019 for PM_2.5_ was higher than in April 2020.

Although many studies have reported improvement in ambient air quality due to the COVID-19 lockdown, it is unknown whether these improvements have occurred across the whole country because these studies were restricted to a specific place or region (e.g., Gautam *et al.* in Kathmandu and Dhobi in Western Terai)^5,6^. In an attempt to represent the air quality of the entire country, the present study included six major cities from six out of seven provinces of Nepal to study the change in PM_2.5_ and PM_10_ concentrations due to lockdown which will help generalize the effect of anthropogenic activities on air quality and be beneficial for environmental, governmental and other related agencies to make effective strategies for pollution control.

## Methods

**Figure 1:**
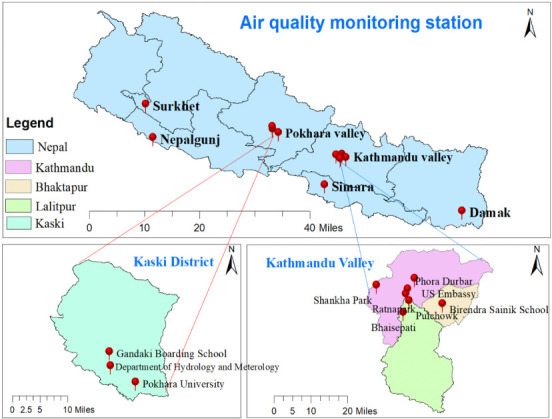
Study area showing the air quality monitoring stations in selected cities. Source: Created by the author using ArcGIS 10.7 software.

For the present study, 6 different cities from 6 out of 7 provinces of Nepal were selected. No city from the 7^th^ province was included due to the unavailability of data from that area. A detailed description of study sites with geographical coordinates of the air quality monitoring center in selected cities is given in Table 1 and their location in Figure 1.

**Table 1:** Study Sites

**Cities**	**Province No.**	**Address of the monitoring station**	**No of station monitored**	**Geographical coordinates**
**Damak**	1	Damak, Jhapa	1	26.669363 N, 87.703262 E

**Simara**	2	Pipara Simara, Bara	1	27.156708 N, 84.997761 E

**Kathmandu Valley**	3	Ratnapark, Kathmandu	7	27.7 N, 85.31 E
		US Embassy, Kathmandu		27.738703 N, 85.336205 E
		Phora Durbar, Kathmandu		27.712463 N, 85.315704 E
		Shankha Park, Kathmandu		27.722654 N, 85.222836 E
		Birendra Sainik School, Bhaktapur		27.673762 N, 85.417528 E
		Pulchowk, Lalitpur		27.682581 N, 85.318841 E
		Bhaisepati, Lalitpur		27.65311 N, 85.302252 E

**Pokhara**	4	Department of Hydrology and Meteorology, Pokhara	3	28.205817 N, 83.97361 E
		Pokhara University, Pokhara Gandaki Boarding School, Pokhara		28.143122 N, 84.08551 E
				28.258 N, 83.968 E

**Nepalgunj**	5	Adarshanagar, Nepalgunj	1	28.05275 N, 81.6222 E

**Surkhet**	6	Kunathari, Surkhet	1	28.678541 N, 81.472938 E

### Analysis

For the analysis of the effect of the lockdown imposed by the governing authorities on air quality, PM_2.5_ and PM_10_ were selected. The threshold for the emission of PM of the WHO is given in Table 2.

**Table 2:** World Health Organization Guidelines for Particulate Matter^27^

**Particulate Matter**	**Concentration**	**Timely mean**
PM_2.5_	10 μg/m^3^	Annual mean
	25 μg/m^3^	24-hour mean
PM_10_	20 μg/m^3^	Annual mean
	50 μg/m^3^	24-hour mean

The government of Nepal has set and enforced NAAQS guidelines and has a legal obligation to maintain PM emission standards. The details of the threshold maintained by the Nepal Government for PM emissions is given in Table 3.

**Table 3:** National Ambient Air Quality Standard^28^

**Particulate Matter**	**Concentration**	**Timely mean**
PM_2.5_	40 μg/m^3^	24-hour mean
PM_10_	120 μg/m^3^	24-hour mean

### Data source

We received the data from the World Air Quality Index project, China, which is a non-profit project started in 2007.^29^ Its mission is to promote air pollution awareness for citizens and provide unified and world-wide air quality information. The project provides transparent air quality information for more than 130 countries, covering more than 30 000 stations in 2 000 major cities, via these two websites: aqicn.org and waqi.info.^29^ The Department of Environment under the Ministry of Population and Environment is the official government agency responsible for air quality management in Nepal and delivers authenticated data from monitoring stations to many organizations and projects including the World Air Quality Index project. The department had established 22 air quality monitoring stations by the end of April 2020 across Nepal^7^ and measurements (every minute) from the monitoring station are broadcasted through the website www.pollution.gov.np.^30^ According to the Department of Environment (2017), monitoring stations are equipped with Grimm electronic dust monitors (EDM) 180 to measure particulate matter of different sizes. It uses light-scattering technology for the counting of particles. A semiconductor-laser assists as the light source while the particle size analyzer/dust monitor regulates the dust concentration (counts/liter) through the optical light scattering method directly; however, the mass concentration is determined by extrapolation. All the stations are real-time monitoring stations.

### Data collection and interpretation

Data were obtained from the website aqicn.org*.*^29^ To clearly visualize how PM concentrations fluctuated before, during and after the lockdown, data was requested from 24 January 2020 to 21 September 2020 which covers the two months preceding and succeeding the lockdown period. The present study obtained the complete dataset of 24-h average concentrations (μg/m^3^) of PM_2.5_ and PM_10_ for the selected sites. In the cities having more than one air quality monitoring station, the PM concentration of all stations were averaged. Data for some days were missing which might be due to technical damage to the automatic monitoring station. Data analysis was performed using RStudio version 1.3.1093 and the R programming language 4.0.0 which is an integrated development environment for R, a programming language for statistical computing and graphics and freely available from the Comprehensive R Archive Network (CRAN) (https://cran.r-project.org/).^31^ The drop percentage was calculated as discussed by Agarwal *et al.* to determine changes in PM_2.5_ and PM_10_ concentration during different time periods.^9^ Two types of drop percentage were calculated: immediate drop percentage to determine the instant drop in PM concentrations after the lockdown and final drop percentage to determine the PM concentration reduction at the end of the lockdown. The immediate drop percentage was calculated by the difference in average PM_2.5_ and PM_10_ concentrations for 7 days before and 7 days after the lockdown and the final drop percentage was calculated by the immediate difference in average PM_2.5_ and PM_10_ concentration 7 days before the lockdown and the last 7 days after the lockdown was lifted. We performed independent sample Mann–Whitney U testing to test the significance of differences in mean concentrations for each site during the lockdown period (24 March–24 July 2020) and the corresponding period in 2019. Similarly, the significance of differences in mean concentrations between the lockdown period and the period immediately before lockdown (23 January–23 March) was also examined using the same test. A trajectory analysis carried out over Damak, Simara, Kathmandu, Pokhara, Nepalgunj and Surkhet using the National Oceanic and Atmospheric Administration (NOAA) Hybrid Single-Particle Lagrangian Integration Trajectory (HYSPLIT) model (https://ready.arl.noaa.gov/HYSPLIT.php)^32^ to study sources of air mass reaching at six locations. The back trajectories were analyzed for 168 h to track the air mass reaching at the measured site. The HYSPLIT is a good model to show the details of the air mass of a place as discussed in Singh and Chauhan 2020).^8^

### Air quality index calculation

The air quality index (AQI) value for a particular locality enables analysis of air quality levels at specific locations. The United States Environmental Protection Agency (USEPA) calculates the AQI for five major air pollutants, for which national air quality standards have been established to safeguard public health. Equation 1 is used to calculate the AQI from the concentration (μg/m^3^) of PM_2.5_ and PM_10_).^33^

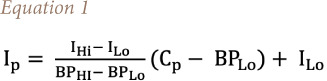
where I_p_ is the index for pollutant p, C_p_ is the truncated concentration of pollutant p, BP_Hi_ is the concentration breakpoint greater than or equal to C_p_, BP_Lo_ is the concentration breakpoint that is less than or equal to C_p_, I_Hi_ is the AQI value corresponding to BP_Hi_, and I_Lo_ is the AQI value corresponding to BP_Lo_.

After calculating the AQI value, it was categorized by level of concern. The USEPA has designated six AQI categories *(Table 4)*, with each category indicating a level of health concern.^33^

**Table 4:** United States Environmental Protection Agency-defined Air Quality Index Categories^33^

**AQI range**	**Level of concern**	**Description of air quality**
0–50	Good	Air quality is satisfactory and air pollution poses little or no risk.
51–100	Moderate	Air quality is acceptable. However, there may be a risk for some people, particularly those who are unusually sensitive to air pollution.
101–150	Unhealthy for sensitive groups	Members of sensitive groups may experience health effects. The general public is less likely to be affected.
151–200	Unhealthy	Some members of the general public may experience health effects; members of sensitive groups may experience more serious health effects.
201–300	Very Unhealthy	Health alert: The risk of health effects is increased for everyone.
301–500	Hazardous	Health warning of emergency conditions: everyone is more likely to be affected.

## Results

**Figure 2:**
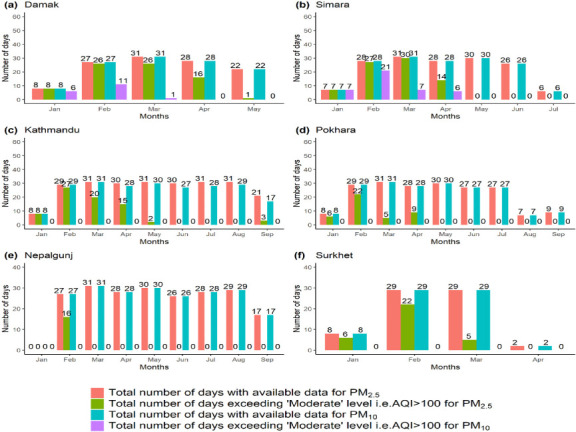
(a), (b), (c), (d), (e) and (f) shows the number of days with available PM_2.5_ and PM_10_ data and the number of days exceeding the USEPA standard^33^ i.e., AQI (PM_2.5_, PM_10_) >100 for the stations at Damak, Simara, Kathmandu, Pokhara, Nepalgunj and Surkhet, respectively.

The AQI_PM2.5_ and AQI_PM10_ were calculated from the average daily recorded values of PM_2.5_ and PM_10_ concentrations before, during and after the lockdown for cities in the present study as shown in Figure 2.

Figure 2 presents the total number of days with available daily PM_2.5_ and PM_10_ data and number of days which exceeded the USEPA ‘moderate category', i.e., AQI>100, for different cities in Nepal. In January and February, the AQI_PM2.5_ level at Damak, Simara, Kathmandu, Pokhara, Nepalgunj and Surkhet exceeded the USEPA standard (moderate level, AQI>100) for the maximum number of days, while after the lockdown, i.e., March 24 2020 onwards, the number of days during which the moderate zone was surpassed began to decrease.

The AQI_PM10_ value exceeded the USEPA ‘moderate category' (AQI>100) for the maximum number of days in Damak and Simara. But after the lockdown, the AQI_PM10_ of these two cities remained within the moderate zone for the maximum number of days. In other cities except Damak and Simara, the AQI_PM10_ remained within the moderate category during the study period.

### Particulate matter

**Figure 3:**
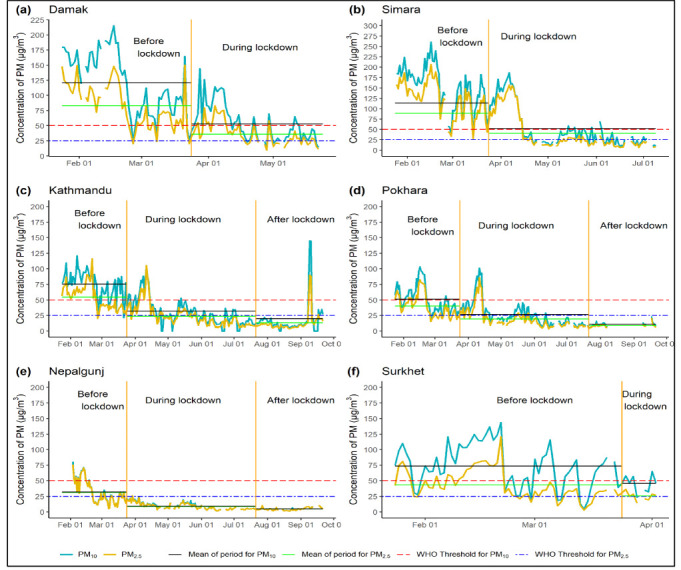
(a), (b), (c), (d), (e) and (f) shows the daily concentrations of PM_2.5_ and PM_10_ from January 24 to September 21, 2020 for the cities of Damak, Simara, Kathmandu, Pokhara, Nepalgunj and Surkhet. (Data for some cities for the specified time are missing due to technical damage to monitoring stations.)

The nationwide lockdown decreased anthropogenic activities, which resulted in a significant drop in PM concentrations (PM_2.5_, PM_10_) in all cities of Nepal (*Figure 3*).

Figure 3 represents the concentration of PM_2.5_ and PM_10_ (μg/m^3^) in different cities of Nepal, before, during and after the lockdown was lifted. Figure 3 also shows the WHO concentration threshold of 25 μg/m^3^ for PM_2.5_ and 50 μg/m^3^ for PM_10_, with values greater than this threshold signifying unhealthy air.^27^

After the lockdown was implemented on March 24 2020, a drastic drop was recorded in mean and daily concentrations of PM_2.5_ and PM_10_ in all cities of Nepal as shown in Figure 3 and summarized in Table 5. Similarly, during and after the lockdown the daily concentrations of PM_2.5_ and PM_10_ remained within the WHO limit (25 μg/m^3^ and 50 μg/m^3^, respectively) for the maximum number of days compared to before the lockdown.

**Table 5:** Variation in PM_2.5_ and PM_10_ Concentrations Before, During and after Lockdown

	**Mean concentration (μg/m^3^)**						**Maximum daily concentration (μg/m^3^)**					
**Cities**	Before lockdown		During lockdown		After lockdown		Before lockdown		During lockdown		After lockdown	
	PM2.5	PM10	PM2.5	PM10	PM2.5	PM10	PM2.5	PM10	PM2.5	PM10	PM2.5	PM10
**Damak**	82.98	121.27	36.06 (46.92)	52.54 (68.73)	-	-	149.8	200.2	75.6 (74.2)	144.3 (55.9)	-	-
**Simara**	89.15	113.27	40.60 (48.55)	52.20 (61.07)	-	-	188.7	260.5	155.6 (33.1)	186.4 (74.1)	-	-
**Kathmandu**	54.40	75.17	24.03 (30.37)	31.76 (43.41)	13.26 (10.77)	19.77 (11.99)	116.28	120.57	105.16 (11.12)	104.17 (16.4)	89.66 (15.5)	144.93 (−40.76)
**Pokhara**	40.07	51.16	19.37 (20.7)	26.63 (24.53)	8.72 (10.65)	10.76 (15.87)	82.37	103.03	85.55 (−3.18)	101.05 (1.98)	21 (64.55)	23.6 (77.45)
**Nepalgunj**	31.01	32.01	8.71 (22.3)	9.29 (22.72)	31.01 (−22.3)	32.01 (−22.72)	76.2	80.6	25.9 (50.3)	24.3 (56.3)	17.3 (8.6)	17.8 (6.5)
**Surkhet**	43.39	73.57	25.67 (17.72)	45.47 (28.1)	-	-	121	143.5	36.2 (84.8)	57.9 (85.6)		

^*^ Figure inside parenthesis indicates change in PM emission from the respective prior period. A negative value indicates increase in PM emissions.

Damak had mean concentrations of PM_2.5_ and PM_10_ of 82.98 μg/m^3^ and 121.27 μg/m^3^, respectively, before the lockdown, dropping to 36.06 μg/m^3^ and 52.54 μg/m^3^, respectively, during the lockdown. Similarly, before the lockdown the maximum daily PM_2.5_ and PM_10_ concentrations were 149.8 μg/m^3^ and 200.2 μg/m^3^, and during the lockdown PM_2.5_ and PM_10_ were 75.6 μg/m^3^ and 144.3 μg/m^3^, respectively *(Table 5).*

The daily concentration of PM_2.5_ and PM_10_ in Damak exceeded the WHO limit 25 μg/m^3^ and 50 μg/m^3^, respectively, for the maximum number of days before the lockdown, and during and after the lockdown remained within the WHO limit (*Figure 3a*). In Simara, Kathmandu, Pokhara, Nepalgunj and Surkhet, a similar pattern was observed with a drastic drop in mean and maximum daily concentration of PM_2.5_ and PM_10_ and daily emissions lower than the WHO limit during and after the lockdown. Data on PM emissions after lockdown in Damak, Simara and Surkhet could not be calculated due to unavailability of this data.

### Air mass trajectory

An analysis of air mass trajectory was performed for sixteen different time periods. Figure 4 shows air mass back trajectories at six locations during 15–21 March and 22–28 March, 15–21 July and 22–28 July for the years 2019 and 2020. The back trajectories provide details about air mass sources at these six measurement locations.

**Figure 4:**
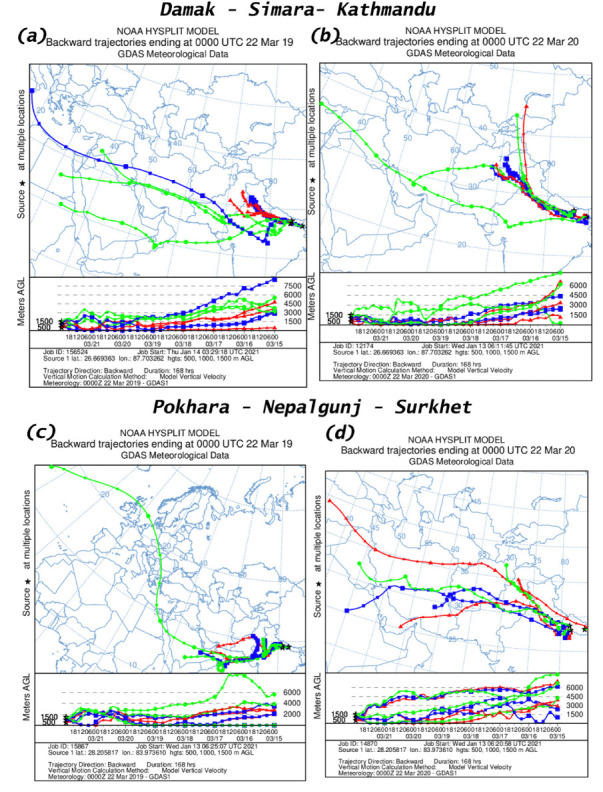
HYSPLIT back trajectory over Damak, Simara, Kathmandu, Pokhara, Nepalgunj and Surkhet during different time periods; (a), (c) 15–21 March 2019; (e), (g) 22–28 March 2019; (i), (k) 15–21 July 2019; (m), (o) 22–28 July 2019 and (b), (d) 15–21 March 2020; (f), (h) 22–28 March 2020; (j), (l) 15–21 July 2019; (n), (p) 22–28 July 2020.

**Figure 4:**
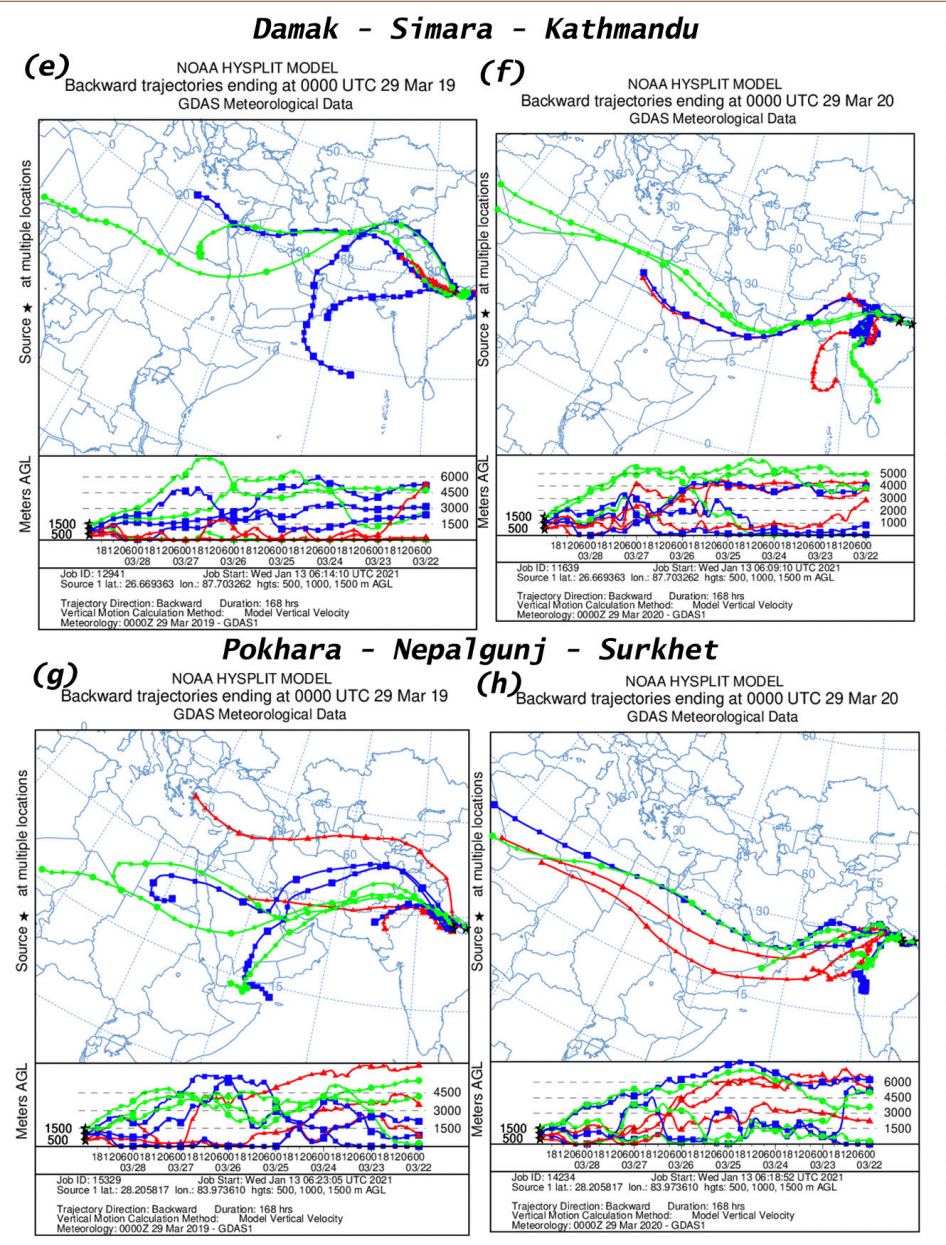
HYSPLIT back trajectory over Damak, Simara, Kathmandu, Pokhara, Nepalgunj and Surkhet during different time periods; (a), (c) 15–21 March 2019; (e), (g) 22–28 March 2019; (i), (k) 15–21 July 2019; (m), (o) 22–28 July 2019 and (b), (d) 15–21 March 2020; (f), (h) 22–28 March 2020; (j), (l) 15–21 July 2019; (n), (p) 22–28 July 2020.

**Figure 4:**
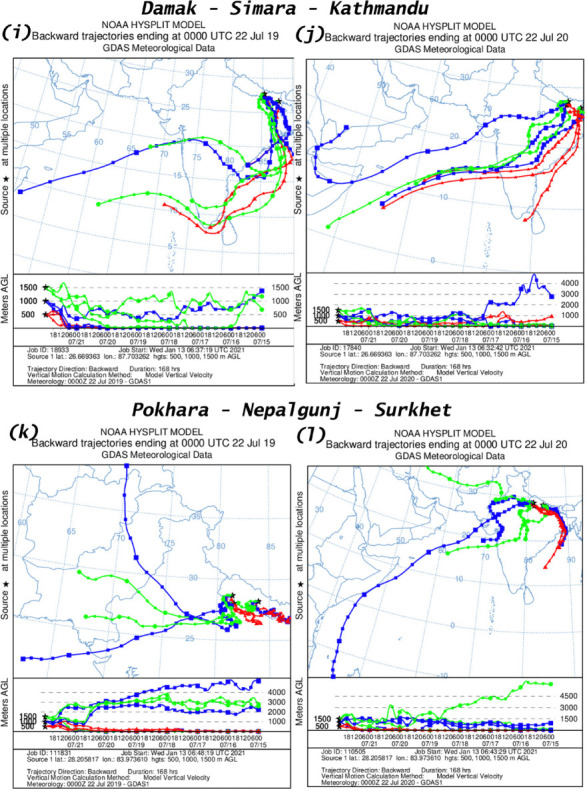
HYSPLIT back trajectory over Damak, Simara, Kathmandu, Pokhara, Nepalgunj and Surkhet during different time periods; (a), (c) 15–21 March 2019; (e), (g) 22–28 March 2019; (i), (k) 15–21 July 2019; (m), (o) 22–28 July 2019 and (b), (d) 15–21 March 2020; (f), (h) 22–28 March 2020; (j), (l) 15–21 July 2019; (n), (p) 22–28 July 2020.

**Figure 4:**
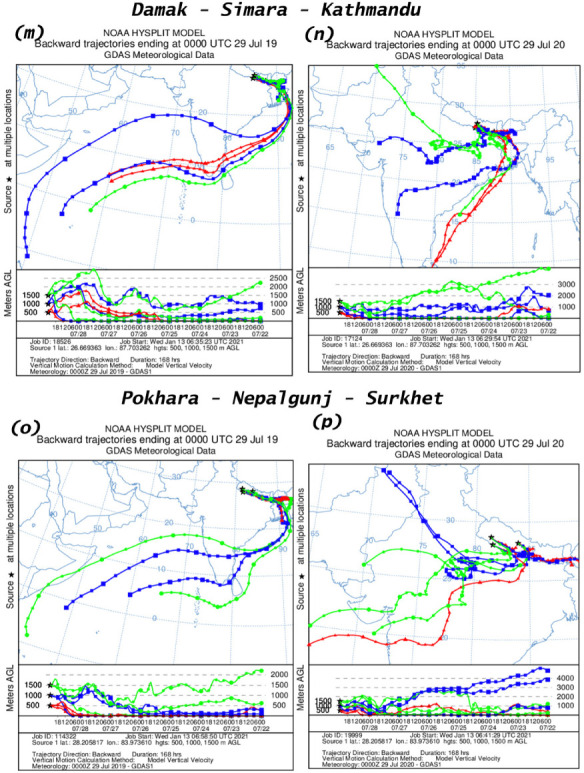
HYSPLIT back trajectory over Damak, Simara, Kathmandu, Pokhara, Nepalgunj and Surkhet during different time periods; (a), (c) 15–21 March 2019; (e), (g) 22–28 March 2019; (i), (k) 15–21 July 2019; (m), (o) 22–28 July 2019 and (b), (d) 15–21 March 2020; (f), (h) 22–28 March 2020; (j), (l) 15–21 July 2019; (n), (p) 22–28 July 2020.

The present study observed that Damak is mostly influenced by a western air mass from Syria during 15–21 March 2019 and during 15–21 March 2020, the air mass originated from the Mediterranean Sea (*Figures 4a–b*). Similarly, during 22–28 March 2019 the air mass of Damak originated from the Arabian Sea and eastern India, while during the corresponding time in 2020 the air mass originated from the Arabian Sea and the Bay of Bengal (*Figures 4e–f*). The air mass of Damak during 15–21 July 2019 originated from the Indian Ocean and the Gulf of Mannar and during the equivalent time in 2020 the air mass originated from the Indian Ocean (*Figures 4i–j*). This pattern was similar to the period of 22–28 July 2019 and the corresponding period of 2020 as the air mass was chiefly received from the Indian Ocean and the Bay of Bengal (*Figures 4m–n*).

Similarly, the sources of air mass reaching to the other cities of Simara, Kathmandu, Pokhara, Nepalgunj, Surkhet before and during the lockdown period and the corresponding period in 2019 are summarized in Table 6 and Figure 4.

**Table 6:** Source of Air Mass Reaching to Different Cities of Nepal

**Cities**	**15–21 March 2019**	**15–21 March 2020**	**22–28 March 2019**	**22–28 March 2020**
**Damak**	Syria	Mediterranean Sea	Arabian Sea and eastern India	Arabian Sea and the Bay of Bengal
**Simara**	The Egyptian desert	Iran	Egyptian desert	Egyptian desert
**Kathmandu**	Italy and the Sahara Desert	Kazakhstan	Mali	Mali
**Pokhara**	The Saudi Arabian desert and the North Atlantic Ocean	Turkey	The Gulf of Aden	Mali
**Nepalgunj**	Saudi Arabia	Saudi Arabia	The Sahara Desert	The Sahara Desert
**Surkhet**	Gulf of Oman and the Persian Gulf	Gulf of Oman and the Persian Gulf	The Sahara Desert	The Arabian Sea

### Immediate and final drop in particulate matter concentrations

Due to the nationwide lockdown, a significant drop was observed in PM_2.5_ and PM_10_ concentrations (μg/m^3^) in almost all cities in Nepal. Table 7 shows the immediate and final drop percentage of PM_2.5_ and PM_10_ concentration across Nepal. The highest immediate drop in PM_2.5_ was observed in Damak (26.37%) and highest final drop in Nepalgunj (80.86%), respectively. Similarly, the highest immediate and final drop of PM_10_ was observed in Surkhet (37.22%) and Nepalgunj (81.14%), respectively. In Pokhara, the immediate drop in both PM_2.5_ and PM_10_ was −15.71% and −8.54% respectively. The negative drop percentage indicates a rise in PM concentration. The final drop percentage of Damak, Simara and Surkhet could not be calculated due to insufficient data.

**Table 7:** Immediate and Final Drop Percentage of PM_2.5_ and PM_10_ Concentrations

**Cities**	**PM25**		**PM10**	
	Immediate drop %	Final drop %	Immediate drop %	Final drop %
**Damak**	26.37	-	12.01	-
**Simara**	−3.48	-	5.37	-
**Kathmandu**	2.45	73.3	27.16	76.4
**Pokhara**	−15.71	71.36	−8.54	73.33
**Nepalgunj**	19.41	80.86	20.13	81.14
**Surkhet**	18.52	-	37.22	-

### Mann Whitney U-test

Analysis with the Mann-Whitney U test indicated that both PM types showed statistically significant (p < 0.05) differences in mean concentration across all sites during lockdown and the corresponding period in 2019. In addition, a significant difference in mean concentrations was observed between the lockdown period and the period immediately before the lockdown. Due to unavailability of data, differences between mean PM concentrations during the pre-lockdown period in 2020 and the corresponding period of 2019 could not be tested.

## Discussion

The COVID-19 lockdown resulted in a significant decrease in PM concentrations in cities across Nepal, largely caused by the reduction of anthropogenic activities and traffic. Our results are in agreement with a study done by Gautam *et al. (2020)* which reported a decrease in AQI_PM10_ and the number of days exceeding the USEPA standard after March 2020 in Kathmandu.^6^ In Pakistan, the AQI_PM2.5_ was observed to improve to normal from the unhealthy to moderate range during lockdown due to termination of economic activities, such as the closure of factories in hotspot zones to prevent further COVID-19 outbreaks.^11^ The results of these studies show that for the maximum number of days, the overall AQI in Nepal remained within the moderate category (AQI<100) during lockdown, highlighting the significant improvements in air quality during this time period.

Before enforcement of the nationwide lockdown on March 24 2020, daily PM_2.5_ and PM_10_ levels in all cities of Nepal exceeded the WHO limit (25 μg/m^3^ and 50 μg/m^3^, respectively, for PM_2.5_ and PM_10_) and the USEPA's moderate category (AQI>100) for the maximum number of days.^33^ After lockdown was enforced there was a significant drop in daily PM concentrations. Studies by Singh & Chauhan (2020), Lian *et al.* (2020), and Hashim *et al.* (2020) in Iraq, China and India, respectively, also reported substantial decreases in daily PM concentrations after the lockdown was imposed.^8,10,12^

After the lockdown was imposed, concentrations of PM_2.5_ and PM_10_ immediately started to decrease in most cities in the present study, but this trend lasted for only a few days after which concentrations rose again, exceeding the WHO limit until mid-April. The period during which emissions began to rise again corresponds with the resuming of vehicular traffic. At that time, the Government of Nepal allowed limited vehicles to transport people who were trapped in the Kathmandu valley back to their home districts and this operation lasted until mid-April.^34,35^ This might be the reason for the continued increase in daily PM_2.5_ and PM_10_ concentrations even after the lockdown enforcement in Damak, Simara, Kathmandu, Pokhara and Surkhet. Nepalgunj is the only city which showed a continuous reduction in PM_2.5_ and PM_10_ concentrations after the lockdown.

Nepalgunj recorded the highest final drop in average PM_2.5_, which might be due to fewer registered vehicles. According to the report of Department of Transport Management (2017), the order of cities from the highest to lowest number of registered vehicles is Kathmandu, Simara, Pokhara, Damak and Nepalgunj.^36^ Despite having the highest number of vehicles, Kathmandu recorded the highest drop in average PM_10_ concentrations. This is because the major source of PM_10_ emissions in Kathmandu involves brick kilns,^37^ which were ordered to cease operations with the lockdown. On the other hand, Kathmandu also had the lowest drop in average PM_2.5_ concentrations which might be due to its geographic valley shape, where recently emitted PM_2.5_ remains in the environment for a longer time.^38^ The lowest drop in average PM_2.5_ and PM_10_ was seen in Pokhara as there was continuous operation of many essential manufacturing industries and even some non-essential industries.^39^ A negative drop percentage was observed in Pokhara and Simara which indicates a rise in PM concentration which might be due to the continuous operation of factories and vehicles for essential purposes during the lockdown. The average PM concentration in Surkhet could not be calculated due to data unavailability.

The HYSPLIT analysis clearly indicated the influence of long-range transport of air masses over the studied cities. The westerly air mass brings dust that affects air quality (PM_2.5_ and AQI) in Nepalgunj and Surkhet, and dust is further transported to the eastern region.^8^ This might explain the higher concentrations of PM in Damak and Simara even during the lockdown period. These results show that the air mass reaching to the studied cities before and after the lockdown was more or less from a similar location. This provides evidence that the major air mass during the study time came from the same source and that meteorological factors had minimal effects on PM emission concentrations in the studied cities.

The average PM_2.5_ and PM_10_ concentration drop during lockdown in Nepal is comparable to the drop percentage recorded in other countries (e.g. Hashim *et al. (2020)* in Iraq; Lian *et al. (2020)* in China; Singh & Chauhan (2020) in India).^8,10,12^ In Iraq, 8% and 15% decreases in PM_2.5_ and PM_10_ concentrations, respectively, were recorded during the first partial and total lockdown, while during a second partial lockdown, PM_2.5_ concentrations decreased by 2.5% and PM_10_ increased by 56%.^12^ There was an immediate 40.2% and 36.86% drop in PM_10_ and PM_2.5_, respectively, in Wuhan city.^10^ Singh and Chauhan (2020) recorded 34.52%, 27.57%, 19.25%, 5.40% and 3.99% immediate drops in daily PM_2.5_ concentrations in Kolkata, Delhi, Mumbai, Chennai and Hyderabad, respectively.^8^ A similar result was obtained in Bangladesh, with a 40% and 32% decrease in PM_2.5_ and PM_10_ concentrations, respectively.^40^ We could not assess the air quality for any city from Province number 7.

## Conclusions

Air pollution is one of the prime factors behind increasing human morbidity and mortality. To the best of our knowledge, the present study is the first investigation to be published analyzing the effect of the COVID-19 lockdown on PM emissions in Nepal. These emissions showed a significant reduction after the lockdown began. More specifically, for the maximum number of days in Nepalgunj, AQI_PM2.5_ and AQI_PM10_ remained within the USEPA standard and also recorded the lowest mean PM emissions during lockdown. Similarly, the highest final drop percentage was observed in Nepalgunj, which showed the biggest improvement in air quality. It shows positive correlation between the number of vehicles and PM emissions, as Nepalgunj had the least number of vehicles. The lower the number of vehicles, the lower the emissions. This shows the importance of regulating the number of vehicles operating in a particular space at one time. This can be best achieved by encouraging people to use public transportation and limiting the use of private vehicles. Similarly, use of energy sources such as compressed natural gas, solar energy and electricity should be promoted as alternatives to fossil fuels to reduce emissions.
